# Trends of HIV-Related Cancer Mortality between 2001 and 2018: An Observational Analysis

**DOI:** 10.3390/tropicalmed6040213

**Published:** 2021-12-20

**Authors:** Chinmay Jani, Omar Al Omari, Harpreet Singh, Alexander Walker, Kripa Patel, Christian Mouchati, Amr Radwan, Zuha Pandit, Georgina Hanbury, Conor Crowley, Dominic C. Marshall, Richard Goodall, Joseph Shalhoub, Justin D. Salciccioli, Umit Tapan

**Affiliations:** 1 Department of Medicine, Mount Auburn Hospital/Beth Israel Lahey Health, Cambridge, MA 02138, USA; omar.alomari@mah.harvard.edu (O.A.O.); alexander.walker@mah.harvard.edu (A.W.); zuha.pandit@mah.harvard.edu (Z.P.); 2 Harvard Medical School, Boston, MA 02115, USA; justin.salciccioli@gmail.com; 3 MDR Collaboration, London W2 1NY, UK; drhpsingh101@gmail.com (H.S.); kripa2493@gmail.com (K.P.); cmouchati@gmail.com (C.M.); georgiehanbury@gmail.com (G.H.); crowconor@gmail.com (C.C.); dominic.marshall@bath.edu (D.C.M.); r.goodall@imperial.ac.uk (R.G.); j.shalhoub@imperial.ac.uk (J.S.);; 4 Division of Pulmonary and Critical Care Medicine, Medical College of Wisconsin, Milwaukee, WI 53226, USA; 5 Smt NHL Municipal Medical College, Ahmedabad 380006, Gujarat, India; 6 Department of Pediatrics, Case Western Reserve University, Cleveland, OH 44106, USA; 7 Department of Pediatrics, University Hospitals Cleveland Medical Center, Cleveland, OH 44106, USA; 8 Department of Internal Medicine, Section of Hematology and Oncology, Boston Medical Center, Boston, MA 02118, USA; amr.elsamanradwan@bmc.org (A.R.); umit.tapan@bmc.org (U.T.); 9 Department of Medicine, Boston University School of Medicine, Boston, MA 02118, USA; 10 Imperial College Healthcare NHS Trust, London W2 1NY, UK; 11 Division of Pulmonary and Critical Care, Lahey Hospital, Burlington, MA 01805, USA; 12 National Heart and Lung Institute, Imperial College London, London SW3 6LY, UK; 13 Department of Surgery and Cancer, Imperial College London, London SW7 2AZ, UK; 14 Imperial Vascular Unit, Imperial College Healthcare NHS Trust, London W12 0HS, UK; 15 Department of Pulmonary and Critical Care, Brigham and Women’s Hospital, Boston, MA 02115, USA

**Keywords:** HIV, mortality, WHO, world, cancer, trend

## Abstract

The burden of AIDS-defining cancers has remained relatively steady for the past two decades, whilst the burden of non-AIDS-defining cancer has increased. Here, we conduct a study to describe mortality trends attributed to HIV-associated cancers in 31 countries. We extracted HIV-related cancer mortality data from 2001 to 2018 from the World Health Organization Mortality Database. We computed age-standardized death rates (ASDRs) per 100,000 population using the World Standard Population. Data were visualized using Locally Weighted Scatterplot Smoothing (LOWESS). Data for females were available for 25 countries. Overall, there has been a decrease in mortality attributed to HIV-associated cancers among most of the countries. In total, 18 out of 31 countries (58.0%) and 14 out of 25 countries (56.0%) showed decreases in male and female mortality, respectively. An increasing mortality trend was observed in many developing countries, such as Malaysia and Thailand, and some developed countries, such as the United Kingdom. Malaysia had the greatest increase in male mortality (+495.0%), and Canada had the greatest decrease (−88.5%). Thailand had the greatest increase in female mortality (+540.0%), and Germany had the greatest decrease (−86.0%). At the endpoint year, South Africa had the highest ASDRs for both males (16.8/100,000) and females (19.2/100,000). The lowest was in Japan for males (0.07/100,000) and Egypt for females (0.028/100,000).

## 1. Introduction

The World Health Organization (WHO) defines Acquired Immune Deficiency Syndrome (AIDS) in people living with HIV (PLWH) either clinically (presence of stage 4 AIDS-defining illness) or immunologically (CD4 cell count less than 200 per mm^3^). PLWH are at increased risk of various forms of malignancies [1,2]. In 2019, there were around 38 million individuals with the human immunodeficiency virus (HIV), out of which 25.4 million were on antiretroviral therapy (ART) [3]. Several malignancies, including Kaposi sarcoma, non-Hodgkin lymphoma, and invasive cervical cancer, are known to develop in the setting of more advanced immunodeficiency and are collectively referred to as AIDS-defining cancers (ADCs) [4]. With HIV infection itself as a risk factor, PLWH are also at increased risk of various forms of non-AIDS-defining cancers (NADC) such as lung and liver cancer [5,6]. Compared to the general population, HIV-positive patients have a 1.6–1.7-fold greater risk of developing cancer [7,8].

One plausible explanation for the association between HIV and cancer is the presence of immunosuppression and chronic inflammation caused by viral persistence [9,10]. Low CD4+ cell count is associated with increased cancer risk, especially NADC [11,12]. HIV infection is associated with blockage of tumor necrosis factor, inhibition of cellular apoptosis, as well as cell cycle dysregulation [13,14,15]. Furthermore, impaired DNA repair is more frequent in PLWH [16]. Increased prevalence of coinfection with oncogenic viruses including Epstein Barr Virus (EBV), Human Herpes Virus 8 (HHV-8), Human Papilloma Virus (HPV), and Hepatitis B and C Viruses (HBV, HCV) further exacerbate immune dysregulation [9,10,17]. Compared to the general population, HIV patients were diagnosed with cancer at a younger age and had a worse prognosis due to advanced disease at presentation [18,19]. 

Malignancies have been a significant contributor to mortality in people living with HIV [1,2]. In a multinational collaboration study from 1999 to 2011, the leading cause of death in HIV patients currently on treatment was AIDS-related cancer [20]. In a population-based study conducted in the United States between 2001–2015, cancer-attributable mortality was 386.9 per 100,000 person-years among PLWH. Of these deaths, 9.2% and 5% of deaths were attributed to NADC and ADCs, respectively [4,21]. In France, malignancy was found to cause mortality in 28% of HIV patients receiving ART [21]. Since the development of ART in the early 1990s, these medications have become more accessible, resulting in earlier initiation of treatment and a subsequent decrease in AIDS-related deaths. ADC rates have followed a similar downward trend [22,23]. However, the rate of NADCs in PLWH is increasing and may be due to the increased life expectancy in PLWH [5]. Despite this, there remains a paucity of data to describe trends in HIV-associated cancer mortality. As such, this is the first large-scale observational study assessing the trends of HIV-associated cancer mortality.

This study’s objective was to describe mortality trends for HIV-associated cancers between 2001 and 2018 using the WHO Mortality Database. Our other objectives were to compute the absolute and relative percentage changes of mortality between the start and endpoint, categorized by country and gender.

## 2. Materials and Methods

### 2.1. Data Sources

We utilized the WHO Mortality Database for the member nations whose data was available. Our current study is based on our recent analysis of trends of HIV mortality [24]. The mortality data were extracted in October 2020 with available data from the WHO mortality database from 2001 and 2018 based on the International Classification of Diseases (ICD) version 10 coding system. Countries were categorized based on their WHO-specified regions. We used ICD-10 code B21 for HIV-associated cancers. B21 code includes HIV disease resulting in Kaposi Sarcoma (B21.0), Burkitt Lymphoma (B21.1), other non-Hodgkin Lymphoma (B21.2), other malignant neoplasm of lymphoid, hematopoietic, and related tissue (B21.3), multiple malignant neoplasms (B21.7), other malignant neoplasms (B21.8), and unspecified neoplasm (B21.9) [25]. For inclusion criteria, we first assessed the database for countries with available data. Of the 194 WHO member countries, data on cancer-related mortality in PLWH were available for 118 nations. Based on WHO Mortality Database 2009–2017 completeness data, 107 countries with completeness <20% were excluded in our data review. Completeness in each country is reported based on the coverage of mortality data. WHO estimates coverage by dividing the total number of deaths that have been registered with cause of death information in the vital registration system for a country-year by the total estimated deaths for that year for the national population [26]. We further excluded 76 countries either because of a lack of data for five years or significant breaks in data lasting more than three consecutive years. All countries included in our study had more than 90% completeness of data except Malaysia (51.81%), Republic of Moldova (79.62%), and Thailand (87.23%). We excluded all the countries from our analysis that had less than 50% of data completeness (number of countries: 107). Details of percentage of data completeness of all the countries included in our analysis is reported in the Supplementary Table S1. 

Crude mortality rates were stratified by gender and reported by year. Overall mortality was reported without any age restrictions. We computed age-standardized death rates (ASDRs) per 100,000 population using the World Standard Population and world average age structure for 1998 [27]. According to the World Standard Population, the ASDR was defined as mortality weighted to the distribution of mortality per 5-year age group [27]. This removes the effects of historical events on age structure and controls the differences in age structure in populations, producing age-specific mortality rates and more representative data. The estimated level of coverage for deaths with a recorded cause of death is calculated by actual reporting divided by the estimated mortality rate. Population and birth recording in all countries are specified in the data, per the WHO standard for inclusion in the database [26]. Formal ethical approval was not required as the data were freely available and in a deidentified format.

### 2.2. Statistical Analyses

We computed male and female mortality rates and used LOWESS plots to fit male and female mortality rates using SAS v9.4 (SAS, Cary, NC, USA). We plotted the results of this analysis for visual inspection. Mortality data were missing in a small subset of countries in the WHO mortality database for one or more calendar years. We excluded the countries with missing data of more than three consecutive years. Relative percentage changes (PC) in ASDR were calculated over the observation as [(End ASDR–Start ASDR)/Start ASDR] ∗ 100 for each gender and country as in our previous studies [24,28,29]. We calculated absolute changes (AC) in ASDR over the observation period as crude absolute differences between the first and last data points for the earliest and most recent years available. AC can be used to compare the results with PC, especially in countries with low baseline mortality.

## 3. Results

We included 31 countries from the following WHO regions: Americas, Western Pacific, South East Asia, Europe, Eastern Mediterranean, and Africa. Of these, one country had data available until 2018, fourteen countries had data available until 2017, six until 2016, five until 2015, two until 2014, one until 2013, one until 2007, and one until 2005. Region-wise, the Americas included the United States of America (USA) and Canada; Europe included Israel, Austria, Belgium, Croatia, Denmark, Estonia, Finland, France, Germany, Italy, Kyrgyzstan, Latvia, Netherlands, Norway, Poland, Republic of Moldova, Romania, Serbia, Spain, Sweden, Switzerland, and the United Kingdom. The Western Pacific region included Japan, Malaysia, Australia, and New Zealand. The Eastern Mediterranean region included only Egypt. Thailand was the only country included in the South East Asia region. Africa included only South Africa.

### 3.1. Current HIV-Associated Cancer Mortality in PLWH

**Figure 1:**
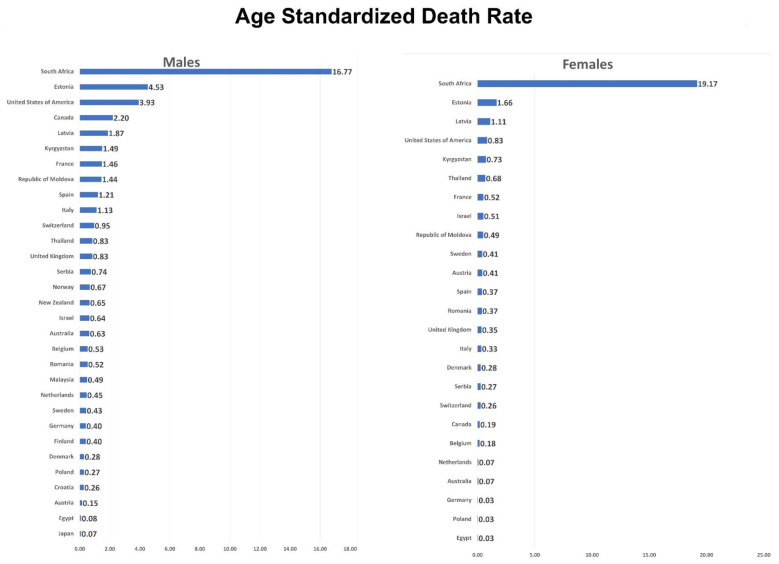
Latest age-standardized death rate in males and females. End point for Canada: 2005, USA: 2007, rest of the countries end point varies between 2013–2018.

Table 1 and Figure 1 show the most recent calendar year mortality data. Among 31 countries having data for males, 25 had available data for females. South Africa had the highest ASDRs for both males (16.8/100,000) and females (19.2/100,000), whereas the lowest ASDR was observed in Japan for males (0.07/100,000) and Egypt for females (0.028/100,000). In the Americas region, the USA had the highest ASDR in 2007 for males (3.9/100,000) and females (0.8/100,000), whereas it was lowest in Canada in 2005 for males (2.2/100,000) as well as for females (0.2/100,000). In Europe, Estonia had the highest ASDR in 2016 for males (4.5/100,000), as well as for females (1.7/100,000), whereas Austria had the lowest ASDR in 2016 for males (0.15/100,000), followed by Croatia (0.26/100,000). Poland had the lowest ASDR in 2017 for females (0.033/100,000), followed by Germany (0.036/100,000). There was not sufficient data available for women for Croatia, Finland, and Norway. In the Western Pacific region, New Zealand had the highest ASDR in 2015 for males (0.65/100,000), whereas Japan had the lowest ASDR in 2017 for males (0.07/100,000). Data for females was only available for Australia, where ASDR in 2017 was 0.07/100,000. In the Eastern Mediterranean region, we only had valid data for Egypt. For males, the last data available in 2013 showed an ASDR of 0.08/100,000, whereas the ASDR for females was 0.028/100,000 in 2015. In Africa, South Africa had an ASDR in 2015 for males of 16.77/100,000 and females of 19.17/100,000. In the Southeast Asia region, Thailand had an ASDR of 0.83/100,000 and 0.68/100,000 for males and females, respectively, in 2017, higher than many other countries in our study group.

### 3.2. Changes in HIV-Associated Cancer Mortality in PLWH between Start and Endpoints

**Figure 2:**
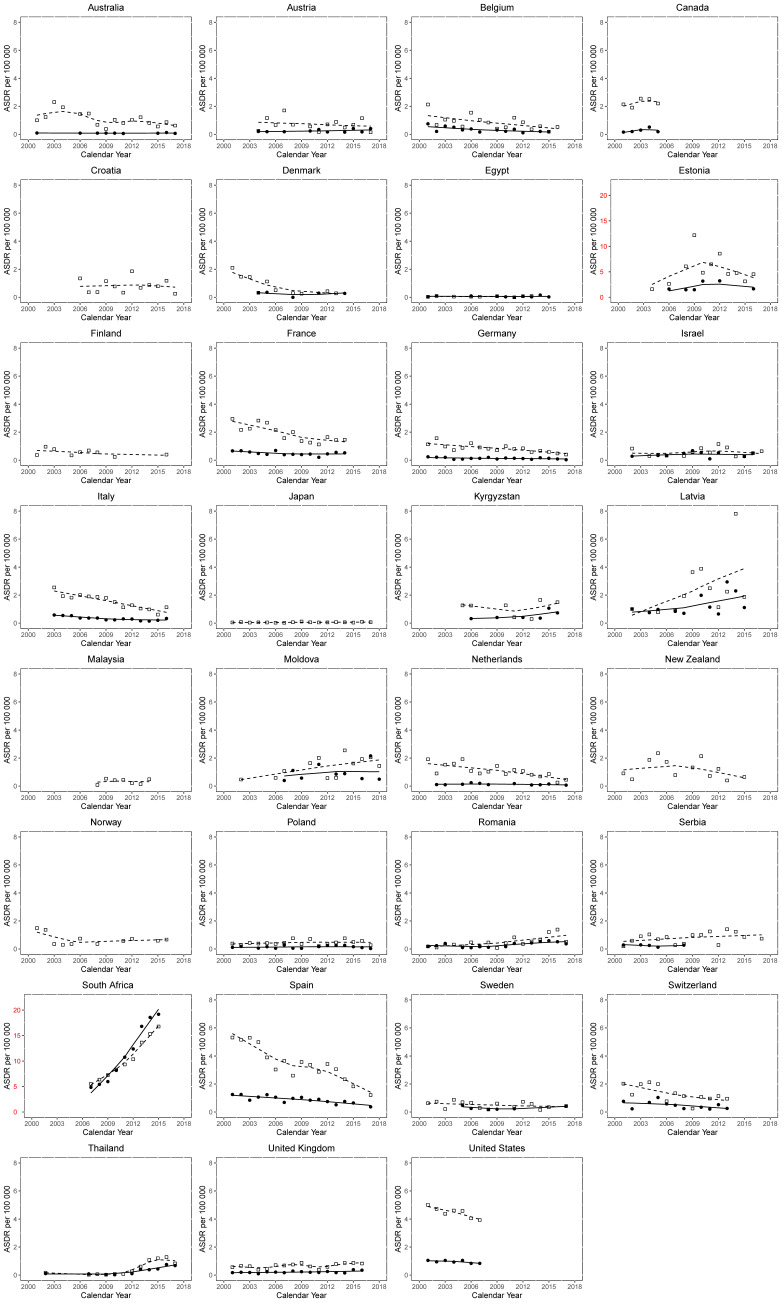
Trends in age-standardized death certification rates per 100,000 for HIV-related cancer. Squares indicate male mortality, whereas circles indicate females. End point for Canada: 2005, USA: 2007, rest of the countries end point varies between 2013–2018.

**Figure 3:**
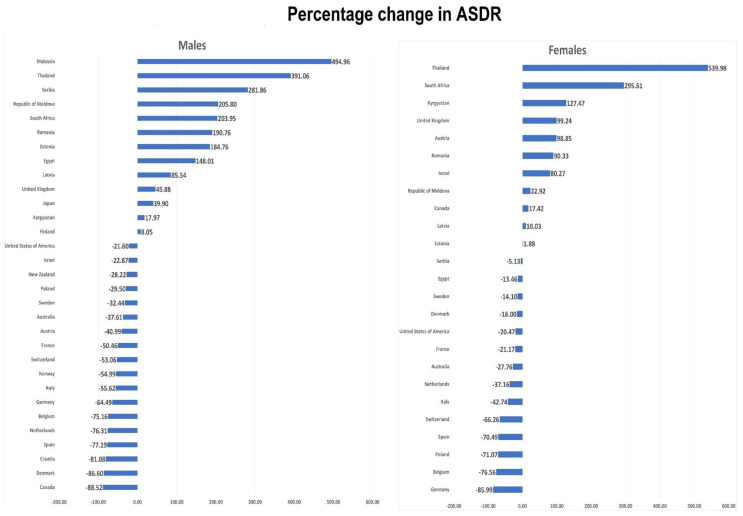
Percentage changes in Age-Standardized Death Rate in males and females between the end and start point of the study. End point for Canada: 2005, USA: 2007, rest of the countries end point varies between 2013–2018.

Trends in ASDRs per 100,000 for HIV-related cancer were assessed for males and females and are represented in Figure 2 using LOWESS plots. Decreasing trends were noted in the majority of countries. Figure 1 and Figure 3 and Table 1 show changes in HIV-associated cancer mortality between the start and end of the observation period. Of 31 countries, 13 (41.9%) showed an increase in the mortality of males, whereas 18 countries (58.0%) showed a decrease in the mortality of males. Of the 25 countries with valid data available for females, 11 countries (44.0%) showed increases in mortality, whereas 14 countries (56.0%) showed a mortality decrease. Among all 31 countries, Malaysia had the highest positive percentage change (PC) in male mortality (+495.0%) between 2008 and 2014, whereas South Africa (+11.25/100,000) had the highest absolute change (AC) between 2007 and 2015. Canada had the greatest negative PC (−88.5%) and AC (−16.97/100,000) in males between 2001 and 2005. Thailand had the greatest positive PC in female mortality (+540.0%) between 2002 and 2017, whereas South Africa had the greatest positive AC (+14.33/100,000) between 2007 and 2015. Germany had the greatest negative PC in female mortality (−86.0%) between 2001 and 2017, whereas Spain had the greatest negative AC (−0.88/100,000) between 2001 and 2017.

In the Americas, the only increase in rate was observed in females in Canada (+17.4%) between 2001 and 2005, whereas males showed a percentage change of −88.5%. In the USA, males had a −21.6% PC, whereas females had a PC of −20.5% between 2001 and 2007. AC are reported in Table 1 for comparison.

In Europe, for males, 14 countries showed decreasing ASDRs during our study period. Serbia, Republic of Moldova, Romania, Estonia, Latvia, United Kingdom, Kyrgyzstan, and Finland increased males’ ASDRs. Of 19 European countries with available ASDR data for females, 11 (57.9%) showed decreasing ASDRs, whereas positive PCs were observed in Kyrgyzstan, the United Kingdom, Austria, Romania, Israel, the Republic of Moldova, Latvia, and Estonia. For males, the highest positive PC was observed in Serbia (+281.9%) between 2001 and 2017, whereas the highest positive AC was observed in Estonia (+2.94/100,000) between 2004 and 2016. For females, the highest positive PC (+127.5%) and AC (+0.41/100,000) were reported in Kyrgyzstan between 2005 and 2016. 

In the Western Pacific region, mortality in males decreased in Australia (PC −37.61%, AC −0.38/100,000) and New Zealand (PC −28.22%, AC −0.26/100,000), whereas it increased in Malaysia (PC +494.96%, AC 0.40/100,000) and Japan (PC +39.9%, AC 0.02/100,000). In females, the only available change was for Australia (PC −27.76%, AC −0.03/100,000) between 2001 and 2017. 

In the Eastern Mediterranean region, we only had PC data available for Egypt. For males between 2001 and 2013, PC and AC were observed as +148% and +0.05/100,000, respectively. At the same time, for females between 2001 and 2015, PC and AC were −15% and −0.01/100,000, respectively.

In Africa, South Africa showed increasing mortality for both males (PC +204.0%, AC +11.25/100,000) and females (PC +295.6%, AC +14.33/100,000).

Thailand showed an increasing mortality in males (PC +391.0%, AC 0.66/100,000) and females (PC +539.98% and AC 0.57/100,000) between 2002 and 2017.

## 4. Discussion

In this observational study of the World Health Organization Mortality Database, HIV-associated cancers decreased in the majority of countries between 2001 and 2018. The decline in cancer-related mortality was seen in 58% (18/31) and 56% (14/25) of the included countries for males and females, respectively. In a small number of countries there were increases in HIV cancer-related mortality including some developing countries such as Malaysia and Thailand as well as in the United Kingdom.

The advent and improved access to ART worldwide have dramatically improved the life expectancy of PLWH, which is now approaching that of the general population. Currently, PLWH represents an ageing group, and malignancies have become a leading cause of morbidity and mortality [20]. Recent studies suggest that PLWH are 60% more likely to develop some form of malignancy than the general population. Moreover, the cancer burden seems to have shifted from ADC to NADC, especially prostate, lung, HCC (hepatocellular carcinoma), NHL (non-Hodgkin lymphoma), and anal SCC (squamous cell carcinoma) [6,7,30]. A recent study from France reported that from 2000 to 2010, ADC and NADC were the cause of death in 10% and 26% of PLWH, respectively [2]. In our study of 31 countries, 13 countries (41.9%) showed an increase in male mortality, and of the 25 countries with valid data available for females, 11 countries (44.0%) showed increases in mortality. Of the countries that had worsening mortality trends, most were in low-income countries, except the UK.

Recent initiatives from the WHO, with the “90-90-90” plan and the “intention to treat” model, have led to more widespread and earlier ART administration in low-income countries. As of the end of June 2020, per UNAIDS, PLWH in upper-middle countries such as South Africa and Thailand were accessing antiretroviral therapy at percentages of 72% and 80%, respectively, compared to the global average of 67% [3]. Despite above-average access to ART, ASDRs and positive PCs were among the highest in these populations, suggesting that the malignancies these patients are developing are not decreasing with widespread ART. The rationale behind the shift is twofold. First, the increased life expectancy in PLWH on ART is now fostering the development of age-associated malignancies [5]. Second, PLWH often have high-risk behaviours that may be risk factors for certain types of malignancy [31]. 

Certain cancers such as breast, cervical, and lung cancers are screened routinely as early detection improves mortality. In PLWH, these malignancies are often found at more advanced stages when compared to the general population, possibly due to a lack of adherence to screening programs [32], or possibly due to under-insurance, low income, and education within this population [33]. Cervical cancer is one of the most common ADCs, and progression of the neoplasm to a malignant state likely reflects both delayed clearance of the Human Papilloma Virus (HPV) for extended periods [34], as well as rapid progression from pre-malignant cervical carcinoma in situ (CIN) to invasive cervical cancer [35]. Specific cervical cancer screening guidelines exist for HIV-infected women [36]. Efforts should be made to increase screening adherence and vaccination for HPV, which is safe and immunogenic in PLWH [37]. The most common NADC is lung cancer, followed closely by liver cancer [5]. According to an American population-based registry, lung cancer is also the most frequent cancer-related cause of death among PLWH. HIV and tobacco use may synergize the risk of pulmonary malignancy as smoking prevalence is higher in PLWH than in the general population [6]. PLWH appear to develop lung cancer 25 to 30 years earlier than the general population. The age of diagnosis in PLWH is between 38 and 57 years, which is significantly below the current USPSTF (the US Preventive services task force) age guideline (50 years) for lung cancer screening [38]. Implementing alternative screening guidelines for PLWH would allow screening at a younger age and might result in the detection of lung cancer at an earlier stage [39,40]. The incidence of liver cancer is also found to be increasing among PLWH. This can be due to coinfection with hepatotoxic viruses such as hepatitis C [41]. High-risk activities, such as increased IVDA (intravenous drug abuse), are more common in some regions of Asia and Africa and likely contribute to the increased HIV prevalence and risk of hepatitis C virus coinfection [42].

Our data demonstrated notable differences in cancer-attributed mortality among males and females. Of 21 countries with valid data available for both sexes, males have higher ASDRs than females in all the countries except South Africa and Austria. Low- and low-middle-income countries, such as Kyrgyzstan and Latvia, had ASDR rate ratios between males and females of 2.05 and 1.68, respectively. High-income countries had similar results, with male mortality being 2.8 and 2.37 times higher in France and the UK, respectively, compared to females. Similar mortality trends in males have been reported elsewhere [43,44]. An epidemiological analysis of cancer-attributable mortality in PLWH in North America found higher mortality in males compared to females (10.2% vs. 7.2%), mainly due to higher mortality attributable to NADCs (7.5% vs. 4.9%) [43]. Males smoked three cigarettes more per day than females, leading to a higher individual risk of cancer [44]. Lower ART compliance might be a contributing factor to higher HIV-associated cancer mortality in males. Data from Malaysia showed that by the end of 2015, about 25,700 (33%) out of 78,000 of PLWH were on ART, with women (70%) having more likelihood of receiving ART compared to men (23%) [45]. 

Research now suggests that PLWH can tolerate cancer treatment and have similar outcomes to the average population, this is with the exception of patients with extremely low CD 4 cell count [46,47]. This emphasizes the need to incorporate PLWH with low CD4 cell counts into clinical trials and guidelines for cancer treatment. The National Cancer Institute sponsors a range of clinical studies in the United States and globally to prevent and treat HIV-associated cancers [48].

There are several limitations of the current investigation that should also be considered when interpreting the results of this study. The WHO mortality database lacks data of incidence and prevalence, and, therefore, our study was limited to the analysis of mortality trends only. Out of 194 countries, data of only 31 countries have been analyzed due to lack of data availability. The starting year varies from 2001 to 2008, and the ending year varies from 2005 to 2018 due to similar reasons (data availability). This can create bias for the results, especially for Canada (end point 2005) and the USA (end point 2007). We were able to include only South Africa from the population of the sub-Saharan region. In 2015, 75.4% of new cases were in sub-Saharan Africa [49]. We understand that this does not represent the entire sub-Saharan population. Further robust reporting is needed from these regions. Variations in cancer-related morbidity are likely to exist; however, this was beyond the remit of this study. Given the observational nature of this study, we do not attempt to attribute causality. As discussed in our previous study, issues exist regarding death certification for HIV [24,50]. However, the WHO has developed computerized coding of the verbal autopsy. Validation studies have shown that 89% of deaths among HIV-positive individuals are attributable to HIV [50]. In terms of strengths, data used in this study were obtained from the accredited WHO Mortality Database. Restrictions on data missingness were applied to increase the robustness of the trend analysis. Standardizing the death rates enabled us to compare patterns in mortality between countries of different characteristics and health systems. We hope the findings of this paper will be of use to future researchers and assist policymakers in the further study of this unique issue of AIDS-associated cancer.

## 5. Conclusions

Overall, there has been a decrease in mortality attributed to HIV-associated cancers in slightly more than half of countries. However, an increasing mortality trend was observed in many developing countries, such as Malaysia and Thailand, and some developed countries, such as the United Kingdom. The exact cause of these specific increases is not clear and further research is required to understand the utility of specific screening methods, early diagnosis, and interventions to reduce cancer-associated mortality in PLWH. 

## Figures and Tables

**Table 1:** Age-Standardized Death Rate during the start and end points of the study.

Sr No	Region	Country	Males						Females					
			Start Point	Age-Standardized Death Rate	End Point	Age-Standardized Death Rate	Percentage Change in Mortality	Absolute Change in Mortality	Start Point	Age-Standardized Death Rate	End Point	Age-Standardized Death Rate	Percentage Change in Mortality	Absolute Change in Mortality
1	Americas	Canada	2001	19.17	2005	2.20	−88.52	−16.97	2001	0.16	2005	0.19	17.42	0.03
2		United States of America	2001	5.01	2007	3.93	−21.60	−1.08	2001	1.05	2007	0.83	−20.47	−0.21
3	Europe	Israel	2002	0.83	2017	0.64	−22.87	−0.19	2002	0.28	2016	0.51	80.27	0.23
4		Austria	2004	0.25	2017	0.15	−40.99	−0.10	2004	0.21	2017	0.41	98.85	0.20
5		Belgium	2001	2.13	2016	0.53	−75.16	−1.60	2001	0.75	2015	0.18	−76.56	−0.57
6		Croatia	2006	1.35	2017	0.26	−81.08	−1.09	NA	NA	NA	NA	NA	NA
7		Denmark	2001	2.11	2013	0.28	−86.60	−1.83	2004	0.33	2014	0.28	−16.00	−0.05
8		Estonia	2004	1.59	2016	4.53	184.76	2.94	2006	1.63	2016	1.66	1.88	0.03
9		Finland	2001	0.37	2016	0.40	8.05	0.03	NA	NA	NA	NA	NA	NA
10		France	2001	2.95	2014	1.46	−50.46	−1.49	2001	0.66	2014	0.52	−21.17	−0.14
11		Germany	2001	1.14	2017	0.40	−64.49	−0.73	2001	0.24	2017	0.03	−85.99	−0.21
12		Italy	2003	2.55	2016	1.13	−55.62	−1.42	2003	0.58	2016	0.33	−42.74	−0.25
13		Kyrgyzstan	2005	1.27	2016	1.49	17.97	0.23	2005	0.32	2016	0.73	127.47	0.41
14		Latvia	2002	1.01	2015	1.87	85.54	0.86	2002	1.01	2015	1.11	10.03	0.10
15		Netherlands	2001	1.92	2017	0.45	−76.31	−1.46	2002	0.11	2017	0.07	−37.16	−0.04
16		Norway	2001	1.49	2016	0.67	−54.99	−0.82	NA	NA	NA	NA	NA	NA
17		Poland	2001	0.39	2017	0.27	−29.50	−0.11	2001	0.11	2017	0.03	−71.07	−0.08
18		Republic of Moldova	2002	0.47	2018	1.44	205.80	0.97	2007	0.40	2018	0.49	22.92	0.09
19		Romania	2001	0.18	2017	0.52	190.76	0.34	2001	0.19	2017	0.37	90.33	0.17
20		Serbia	2001	0.19	2017	0.74	281.86	0.55	2001	0.29	2008	0.27	−5.13	−0.01
21		Spain	2001	5.31	2017	1.21	−77.19	−4.10	2001	1.25	2017	0.37	−70.49	−0.88
22		Sweden	2001	0.63	2017	0.43	−32.44	−0.20	2005	0.48	2017	0.41	−14.10	−0.07
23		Switzerland	2001	2.03	2013	0.95	−53.06	−1.07	2001	0.76	2013	0.26	−66.26	−0.51
24		United Kingdom	2001	0.57	2016	0.83	45.88	0.26	2001	0.17	2016	0.35	99.24	0.17
25	Western Pacific	Japan	2001	0.05	2017	0.07	39.90	0.02	NA	NA	NA	NA	NA	NA
26		Malaysia	2008	0.08	2014	0.49	494.96	0.40	NA	NA	NA	NA	NA	NA
27		Australia	2001	1.00	2017	0.63	−37.61	−0.38	2001	0.10	2017	0.07	−27.76	−0.03
28		New Zealand	2001	0.91	2015	0.65	−28.22	−0.26	NA	NA	NA	NA	NA	NA
29	Eastern Mediterranean	Egypt	2001	0.03	2013	0.08	148.01	0.05	2001	0.033	2015	0.028	−15.15	−0.01
30	Southeast Asia	Thailand	2002	0.17	2017	0.83	391.06	0.66	2002	0.11	2017	0.68	539.98	0.57
31	Africa	South Africa	2007	5.52	2015	16.77	203.95	11.25	2007	4.85	2015	19.17	295.61	14.33

## Data Availability

We extracted HIV-related cancer mortality data from the WHO Mortality Database from 2001 to 2018 for the member countries with available data.

## Supplementary Materials

The following are available online at https://www.mdpi.com/article/10.3390/tropicalmed6040213/s1, Supplementary Table S1: Completeness of data for each country in WHO mortality database.

## References

[1] Palella F.J., Delaney K.M., Moorman A.C., Loveless M.O., Fuhrer J., Satten G.A., Aschman D.J., Holmberg S.D. (1998). Declining morbidity and mortality among patients with advanced human immunodeficiency virus infection. HIV Outpatient Study Investigators. N. Engl. J. Med..

[2] Morlat P., Roussillon C., Henard S., Salmon D., Bonnet F., Cacoub P., Georget A., Aouba A., Rosenthal E., May T. (2014). Causes of death among HIV-infected patients in France in 2010 (national survey): Trends since 2000. AIDS.

[3] Global HIV & AIDS Statistics—2020 Fact Sheet. https://www.unaids.org/en/resources/fact-sheet.

[4] Horner M.J., Shiels M.S., Pfeiffer R.M., Engels E.A. (2020). Deaths attributable to cancer in the United States HIV population during 2001–2015. Clin. Infect. Dis..

[5] Shiels M.S., Pfeiffer R.M., Gail M.H., Hall H.I., Li J., Chaturvedi A.K., Bhatia K., Uldrick T.S., Yarchoan R., Goedert J.J. (2011). Cancer burden in the HIV-infected population in the United States. J. Natl. Cancer Inst..

[6] Engels E.A., Brock M.V., Chen J., Hooker C.M., Gillison M., Moore R.D. (2006). Elevated incidence of lung cancer among HIV-infected individuals. J. Clin. Oncol..

[7] Park L.S., Tate J.P., Sigel K., Rimland D., Crothers K., Gibert C., Rodriguez-Barradas M.C., Goetz M.B., Bedimo R.J., Brown S.T. (2016). Time trends in cancer incidence in persons living with HIV/AIDS in the antiretroviral therapy era: 1997–2012. AIDS.

[8] Hernandez-Ramirez R.U., Shiels M.S., Dubrow R., Engels E.A. (2017). Cancer risk in HIV-infected people in the USA from 1996 to 2012: A population-based, registry-linkage study. Lancet HIV.

[9] Robbins H.A., Pfeiffer R.M., Shiels M.S., Li J., Hall H.I., Engels E.A. (2015). Excess cancers among HIV-infected people in the United States. J. Natl. Cancer Inst..

[10] Hart B.B., Nordell A.D., Okulicz J.F., Palfreeman A., Horban A., Kedem E., Neuhaus J., Jacobs D.R., Duprez D.A., Neaton J.D. (2018). Inflammation-Related Morbidity and Mortality Among HIV-Positive Adults: How Extensive Is It?. J. Acquir. Immune Defic. Syndr..

[11] Dubrow R., Silverberg M.J., Park L.S., Crothers K., Justice A.C. (2012). HIV infection, aging, and immune function: Implications for cancer risk and prevention. Curr. Opin. Oncol..

[12] Engels E.A., Biggar R.J., Hall H.I., Cross H., Crutchfield A., Finch J.L., Grigg R., Hylton T., Pawlish K.S., McNeel T.S. (2008). Cancer risk in people infected with human immunodeficiency virus in the United States. Int. J. Cancer.

[13] Harrod R., Nacsa J., Van Lint C., Hansen J., Karpova T., McNally J., Franchini G. (2003). Human immunodeficiency virus type-1 Tat/co-activator acetyltransferase interactions inhibit p53Lys-320 acetylation and p53-responsive transcription. J. Biol. Chem..

[14] Campioni D., Corallini A., Zauli G., Possati L., Altavilla G., Barbanti-Brodano G. (1995). HIV type 1 extracellular Tat protein stimulates growth and protects cells of BK virus/tat transgenic mice from apoptosis. AIDS Res. Hum. Retrovir..

[15] Colombrino E., Rossi E., Ballon G., Terrin L., Indraccolo S., Chieco-Bianchi L., De Rossi A. (2004). Human immunodeficiency virus type 1 Tat protein modulates cell cycle and apoptosis in Epstein-Barr virus-immortalized B cells. Exp. Cell Res..

[16] Wistuba I.I., Behrens C., Milchgrub S., Virmani A.K., Jagirdar J., Thomas B., Ioachim H.L., Litzky L.A., Brambilla E.M., Minna J.D. (1998). Comparison of molecular changes in lung cancers in HIV-positive and HIV-indeterminate subjects. JAMA.

[17] Shmakova A., Germini D., Vassetzky Y. (2020). HIV-1, HAART and cancer: A complex relationship. Int. J. Cancer.

[18] Cutrell J., Bedimo R. (2013). Non-AIDS-defining cancers among HIV-infected patients. Curr. HIV/AIDS Rep..

[19] Ipp H., Zemlin A.E., Erasmus R.T., Glashoff R.H. (2014). Role of inflammation in HIV-1 disease progression and prognosis. Crit. Rev. Clin. Lab. Sci..

[20] Smith C.J., Ryom L., Weber R., Morlat P., Pradier C., Reiss P., Kowalska J.D., de Wit S., Law M., el Sadr W. (2014). Trends in underlying causes of death in people with HIV from 1999 to 2011 (D:A:D): A multicohort collaboration. Lancet.

[21] Bonnet F., Lewden C., May T., Heripret L., Jougla E., Bevilacqua S., Costagliola D., Salmon D., Chene G., Morlat P. (2004). Malignancy-related causes of death in human immunodeficiency virus-infected patients in the era of highly active antiretroviral therapy. Cancer.

[22] Reniers G., Slaymaker E., Nakiyingi-Miiro J., Nyamukapa C., Crampin A.C., Herbst K., Urassa M., Otieno F., Gregson S., Sewe M. (2014). Mortality trends in the era of antiretroviral therapy: Evidence from the Network for Analysing Longitudinal Population based HIV/AIDS data on Africa (ALPHA). AIDS.

[23] Rufu A., Chitimbire V.T.S., Nzou C., Timire C., Owiti P., Harries A.D., Apollo T. (2018). Implementation of the ‘Test and Treat’ policy for newly diagnosed people living with HIV in Zimbabwe in 2017. Public Health Action.

[24] Jani C., Patel K., Walker A., Singh H., Al Omari O., Crowley C., Marshall D.C., Goodall R., Rupal A., Salciccioli J.D. (2021). Trends of HIV Mortality between 2001 and 2018: An Observational Analysis. Trop. Med. Infect. Dis..

[25] World Health Organization (2014). Annex 2, ICD-10 codes related to HIV. Guidelines for HIV Mortality Measurement.

[26] World Health Organization Completeness of Cause of Death Data.

[27] Ahmad O.B., Boschi-Pinto C., ALopez l.D., Murray C.J.L., Lozano R., Inoue M. (2001). Age Standardization of Rates: A New Who Standard.

[28] Marshall D.C., Salciccioli J.D., Shea B.S., Akuthota P. (2018). Trends in mortality from idiopathic pulmonary fibrosis in the European Union: An observational study of the WHO mortality database from 2001–2013. Eur. Respir. J..

[29] Hartley A., Marshall D.C., Salciccioli J.D., Sikkel M.B., Maruthappu M., Shalhoub J. (2016). Trends in Mortality From Ischemic Heart Disease and Cerebrovascular Disease in Europe: 1980 to 2009. Circulation.

[30] Beral V. (1991). Epidemiology of Kaposi’s sarcoma. Cancer Surv..

[31] Park L.S., Hernandez-Ramirez R.U., Silverberg M.J., Crothers K., Dubrow R. (2016). Prevalence of non-HIV cancer risk factors in persons living with HIV/AIDS: A meta-analysis. AIDS.

[32] Shiels M.S., Copeland G., Goodman M.T., Harrell J., Lynch C.F., Pawlish K., Pfeiffer R.M., Engels E.A. (2015). Cancer stage at diagnosis in patients infected with the human immunodeficiency virus and transplant recipients. Cancer.

[33] Corrigan K.L., Wall K.C., Bartlett J.A., Suneja G. (2019). Cancer disparities in people with HIV: A systematic review of screening for non-AIDS-defining malignancies. Cancer.

[34] Chirenje Z.M. (2005). HIV and cancer of the cervix. Best Pract. Res. Clin. Obstet. Gynaecol..

[35] Rellihan M.A., Dooley D.P., Burke T.W., Berkland M.E., Longfield R.N. (1990). Rapidly progressing cervical cancer in a patient with human immunodeficiency virus infection. Gynecol. Oncol..

[36] Kaplan J.E., Benson C., Holmes K.K., Brooks J.T., Pau A., Masur H., Pau A. (2009). Guidelines for prevention and treatment of opportunistic infections in HIV-infected adults and adolescents: Recommendations from CDC, the National Institutes of Health, and the HIV Medicine Association of the Infectious Diseases Society of America. MMWR Recomm. Rep..

[37] Wilkin T., Lee J.Y., Lensing S.Y., Stier E.A., Goldstone S.E., Berry J.M., Jay N., Aboulafia D., Cohn D.L., Einstein M.H. (2010). Safety and immunogenicity of the quadrivalent human papillomavirus vaccine in HIV-1-infected men. J. Infect. Dis..

[38] Lung Cancer: Screening. https://uspreventiveservicestaskforce.org/uspstf/recommendation/lung-cancer-screening#fullrecommendationstart.

[39] Reddy K.P., Kong C.Y., Hyle E.P., Baggett T.P., Huang M., Parker R.A., Paltiel A.D., Losina E., Weinstein M.C., Freedberg K.A. (2017). Lung Cancer Mortality Associated with Smoking and Smoking Cessation Among People Living With HIV in the United States. JAMA Intern. Med..

[40] Sigel K., Makinson A., Thaler J. (2017). Lung cancer in persons with HIV. Curr. Opin. HIV AIDS.

[41] Cockerham L., Scherzer R., Zolopa A., Rimland D., Lewis C.E., Bacchetti P., Grunfeld C., Shlipak M., Tien P.C. (2010). Association of HIV infection, demographic and cardiovascular risk factors with all-cause mortality in the recent HAART era. J. Acquir. Immune Defic. Syndr..

[42] Thorne C., Ferencic N., Malyuta R., Mimica J., Niemiec T. (2010). Central Asia: Hotspot in the worldwide HIV epidemic. Lancet Infect. Dis..

[43] Engels E.A., Yanik E.L., Wheeler W., Gill M.J., Shiels M.S., Dubrow R., Althoff K.N., Silverberg M.J., Brooks J.T., Kitahata M.M. (2017). Cancer-Attributable Mortality Among People With Treated Human Immunodeficiency Virus Infection in North America. Clin. Infect. Dis..

[44] Tesoriero J.M., Gieryic S.M., Carrascal A., Lavigne H.E. (2010). Smoking among HIV positive New Yorkers: Prevalence, frequency, and opportunities for cessation. AIDS Behav..

[45] Malaysia Country Facts. UNAIDS. https://www.unaids.org/en/regionscountries/countries/malaysia.

[46] Calkins K.L., Chander G., Joshu C.E., Visvanathan K., Fojo A.T., Lesko C.R., Moore R.D., Lau B. (2020). Immune Status and Associated Mortality After Cancer Treatment Among Individuals With HIV in the Antiretroviral Therapy Era. JAMA Oncol..

[47] Bender Ignacio R., Ddungu H., Uldrick T.S. (2020). Untangling the Effects of Chemotherapy and HIV on CD4 Counts-Implications for Immunotherapy in HIV and Cancer. JAMA Oncol..

[48] Halpern S.D., Ubel P.A., Caplan A.L. (2002). Solid-organ transplantation in HIV-infected patients. N. Engl. J. Med..

[49] Collaborators G.H. (2016). Estimates of global, regional, and national incidence, prevalence, and mortality of HIV, 1980-2015: The Global Burden of Disease Study 2015. Lancet HIV.

[50] World Health Organization (2014). Obtaining Cause-of-Death Information for HIV/AIDS. Guidelines for HIV Mortality Measurement.

