# Supplementary material for: Trends of HIV-Related Cancer Mortality between 2001 and 2018: An Observational Analysis
Source: Trop Med Infect Dis. 2021 Dec 20;6(4):213. doi: 10.3390/tropicalmed6040213 (PMC8707967; doi:10.3390/tropicalmed6040213)
Supplement: Supplementary file 1 [file tropicalmed-06-00213-s001.zip › tropicalmed-1446223-supplementary.pdf]

Tropical Medicine and Infectious Disease

MDPI

Supplementary material of Trends of HIV-Related Cancer Mortality between 2001 and 2018: An Observational Analysis

Table S1. Completeness of data for each country in WHO mortality database.

Country	Year	%
Australia	2016	100
Austria	2017	100
Belgium	2016	100
Canada	2015	100
Croatia	2016	100
Denmark	2015	100
Egypt	2015	93.99
Estonia	2016	100
Finland	2016	100
France	2015	100
Germany	2016	100
Israel	2016	100
Italy	2015	100
Japan	2016	100
Kyrgyzstan	2016	91.01
Latvia	2015	100
Malaysia	2014	51.81
Netherlands	2016	100
New Zealand	2014	100
Norway	2016	100
Poland	2016	100
Republic of Moldova	2017	79.62
Romania	2017	100
Serbia	2016	94.05
South Africa	2015	92.47
Spain	2016	100
Sweden	2016	100
Switzerland	2016	100
Thailand	2016	87.23
United Kingdom	2016	100
United States of america	2016	100
